# Gene model for the ortholog of *JhI-26* and a paralog in *Drosophila dunni*

**DOI:** 10.17912/micropub.biology.002272

**Published:** 2026-07-07

**Authors:** Camille Canard, Pablo Chialvo, Clare Scott Chialvo

**Affiliations:** 1 Biology, Appalachian State University, Boone, North Carolina USA

## Abstract

We developed a gene model for the *Juvenile hormone-inducible protein 26* ortholog ( *JhI-26* ) and a directly upstream paralog in the ASM1815212v1 Genome Assembly (GenBank Accession: GCA_018152125.1) of *Drosophila dunni* . This ortholog and its paralog were characterized as part of a developing dataset for a comparative study of detoxification gene family evolution in the *immigrans* - *tripunctata* radiation of the genus *Drosophila* using an adapted Genomics Education Partnership gene annotation protocol for Course-based Undergraduate Research Experiences.

**Figure 1. Genomic neighborhood and gene model for JhI-26 ortholog and paralog in D. dunni:** (A) **Synteny comparison of the genomic neighborhoods for *JhI-26* in *Drosophila melanogaster* and *D. dunni* .** Thin underlying arrows indicate which DNA strand the target gene, *JhI-26* , is located on in *D. melanogaster* (top) and *D. dunni* (bottom). The thin arrow pointing to the right indicates that *JhI-26* is on the positive strand in *D. melanogaster* , and the thin arrow pointing to the right indicates that *JhI-26* is also on the positive strand in *D. dunni* . The wide gene arrows pointing in the same direction as *JhI-26* are on the same strand relative to the thin underlying arrows, while wide gene arrows pointing in the opposite direction of *JhI-26* are on the opposite strand relative to the thin underlying arrows. White gene arrows in *D. dunni* indicate orthology to the corresponding gene in *D. melanogaster* . Other colors of arrows indicate: black = non-orthology, grey = present in both neighborhoods but not syntenic, and blue = target gene duplication. Gene symbols given in the *D. dunni* gene arrows indicate the orthologous gene in *D. melanogaster* , while the locus identifiers are specific to *D. dunni* . (B) **Gene Model in GEP UCSC Track Data Hub** (Raney et al., 2014). The coding-regions of the *JhI-26* ortholog and paralog in *D. dunni* are displayed in the User Supplied Track (red); coding sequences (CDS) are depicted by thick rectangles and introns by thin lines with arrows indicating the direction of transcription. Subsequent evidence tracks include Spaln of *D. melanogaster* Proteins (purple, alignment of Ref-Seq proteins from *D. melanogaster* ), Coding Regions Predicted by Augustus (dark blue), GeMoMa (teal), and NSCAN PASA-EST (dark green), and RNA-Seq from mixed sex adult flies (brown; alignment of mixed sex adult Illumina RNA-Seq reads from *D. dunni* – Erlenbach et al. 2023). (C) **Dot Plot of JhI-26-PA in *D. melanogaster* ( *x* -axis) vs. the orthologous and paralogous peptides in *D. dunni* ( *y* -axis).** Amino acid number is indicated along the left and bottom; CDS number is indicated along the top and right, and CDSs are also highlighted with alternating colors. Line breaks in the dot plot indicate areas of with low sequence identity between species. In the *D. dunni* ortholog, there is one longer break in CDS 3 (dark blue box – a) and one longer break in CDS 4 (light yellow box – b). In the *D. dunni* paralog, there is a long break that spans most of CDS 1 (green box – c), a longer break in CDS 3 (light blue box – d), and one long break in CDS 4 (fuchsia box – e). (D) **Idiosyncrasies in protein alignment.** In the *JhI-26* ortholog and paralog, we note breaks in the protein alignments that indicate low levels of sequence similarity. For CDS 3 of the ortholog there is one longer break in the protein alignment (dark blue box – a), across the break over half of the 61 amino acids are conserved or highly chemically similar. In CDS 4 of the ortholog, the long break (light yellow box – b), covers 80 amino acids with only 16 are very dissimilar and an insertion of three amino acids at the end. In the paralog, long breaks were noted in CDSs 1, 3, and 4. In CDS 1, the long break (green box) spans the full length of the CDS (80 amino acids) and 64 are chemically similar or conserved, 15 are dissimilar, and a single amino acid insertion is found in *D. dunni* . The long break in CDS 3 spans 67 amino acids, but only 15 of these are dissimilar. The break in CDS 4 covers most of the length of this CDS (86 amino acids out of 94 total). Of the amino acids found in this break, 64 are chemically similar or conserved, 18 are dissimilar, and an insertion of four amino acids occurs at the end of the *D. dunni* paralog.

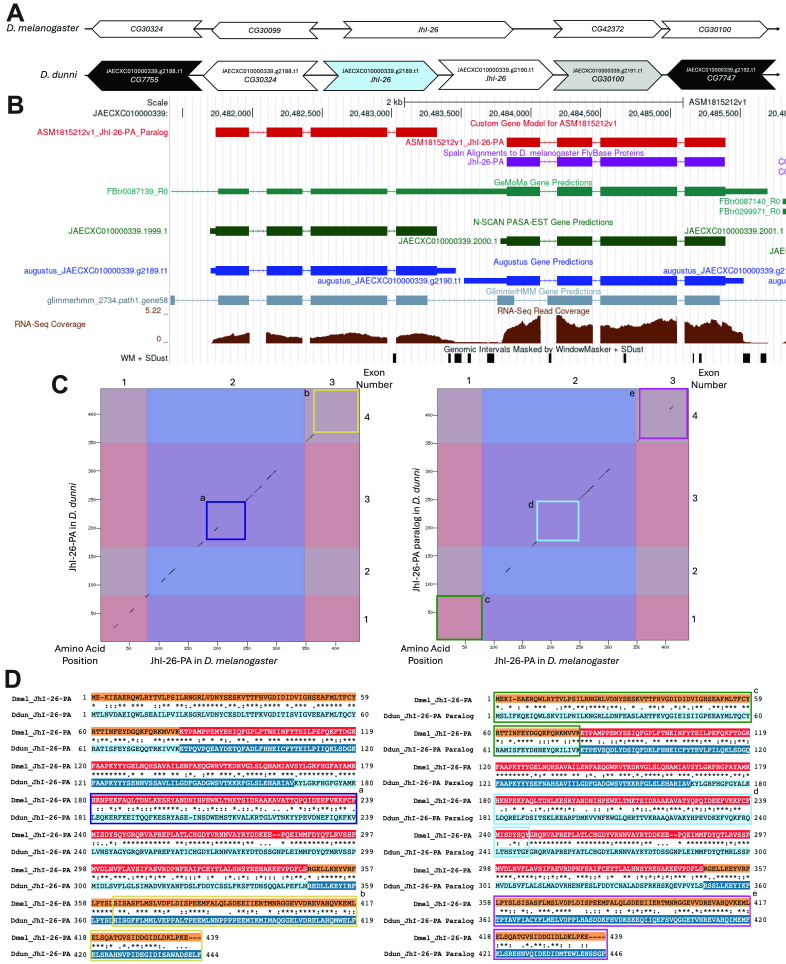



## Description

**Table:** 

* This article reports a predicted gene model generated by undergraduate work using a structured gene model annotation protocol defined by the Genomics Education Partnership (GEP; thegep.org ) for Course-based Undergraduate Research Experience (CURE). The following information in this box may be repeated in other articles submitted by participants using the same GEP CURE protocol for annotating Drosophila species orthologs of Drosophila melanogaster detoxification genes. * “Within insects, the process of detoxifying xenobiotics and host secondary metabolites is a three-phase process that involves functionalization, conjugation, and excretion of these compounds. Expansions of known detoxification gene families ( *e.g.* , cytochrome P450s) is associated with diet breadth and insecticide resistance (Ranson et al., 2002; Després et al., 2007; Rane et al., 2016). With the increasing availability of high-quality genomes for non-model organisms, including *Drosophila* species beyond *D. melanogaster* , it is now possible to perform large scale comparative studies (Robinson et al., 2011; Kim et al., 2021; Threfall and Baxter, 2021). Careful manual annotation and curation of gene models can improve upon computational gene predictions in non-model species, which aids the accuracy of studies on gene and genome evolution (Mudge and Harrow, 2016; Tello-Ruiz et al., 2019). To aid in these annotations, the Genomics Education Partnership (thegep.org) developed a curriculum involving web-based tools that allow undergraduates to engage in authentic course-based research focused on manually annotating genes in non-model species (Rele et al., 2023). The orthologous gene models, including the one presented here, then provide a reliable basis for further evolutionary genomic analyses when made available to the scientific community. The gene ortholog and paralog described here in *Drosophila dunni* for *Juvenile hormone-inducible protein 26* ( *JhI-26* ), a member of the ecdysteroid kinase-like (EcKL) gene family, was characterized as part of a developing dataset for a comparative study of detoxification gene families in the *immigrans* - *tripunctata* radiation of the genus *Drosophila* .” (Williams et al., 2026) “Within the subgenus *Drosophila* , *D. dunni* Townsend and Wheeler 1955 is placed in the *dunni* subgroup of the *cardini* species group in the *immigrans-tripunctata* radiation (Heed and Krishnamurthy, 1959; Bächli, 2005). Species in the *dunni* subgroup, including *D. dunni* , are distributed across the Caribbean (Heed and Krishnamurthy, 1959). Members of the *cardini* group primarily feed and develop on fruit and flowers (Markow and O'Grady, 2008). While some mushroom-feeding *cardini* subgroup members tolerate the mushroom toxin α-amanitin (Stump et al., 2011), the fruit/flower feeding *dunni* subgroup species do not (Erlenbach et al., 2023).” (Williams et al., 2026)

The ecdysteroid kinase-like genes (EcKL) are classified as arthropod specific phase II detoxification enzymes (Blum et al., 2020; Scanlan et al., 2020). These gene act by phosphorylating both hormones associated with insect metamorphosis and xenobiotics (Sonobe et al., 2006; Scanlan and Robin, 2024). Although detoxifying compounds through the addition of phosphates is rare in mammals, this method is common in insects and bacteria (Mitchell, 2015; Scanlan et al., 2022). *Juvenile hormone-inducible protein 26* ( *JhI-26* ) is an EcKL gene associated with spermatogenesis and whose expression can be induced by exposure to juvenile hormone during insect development (Dubrovsky et al., 2000; Wasbrough et al., 2010). *Wolbachia* infections lead to an upregulation of *JhI-26* in the testes (Zheng et al., 2011); this is thought to contribute to cytoplasmic incompatibility (Liu et al., 2014). Beyond its role in development, Scanlan et al. (2020) showed that exposure to xenobiotics induces expression of *JhI-26* with a detoxification score of 3 (second highest score).

We propose a gene model for the *D. dunni* ortholog of the *D. melanogaster* *Juvenile hormone-inducible protein 26* ( *JhI-26* ) gene and a paralog that is the first upstream neighbor of the ortholog. The genomic region of the ortholog corresponds to the Augustus gene prediction JAECXC010000339 .g2190.t1 in the ASM1815212v1 Genome Assembly of *D. dunni* ( GCA_018152125.1 – Kim et al., 2021). The paralog corresponds to the JAECXC010000339 .g2189.t1 Augustus prediction. This model is based on mixed sex, adult RNA-Seq data from *D. dunni* (Erlenbach et al. 2023; https://doi.org/10.5061/dryad.hdr7sqvq2 ) and *JhI-26* in *D. melanogaster* using FlyBase release FB2023_01 ( GCA_000001215.4 ; Gramates et al., 2022; Jenkins et al., 2022; Larkin et al., 2021).

***Synteny***

The reference gene, *JhI-26 ,* occurs on the positive strand of chromosome 2R in *D. melanogaster* and is flanked upstream by *CG30099* and *CG30324* and downstream by *CG42372* and *CG30100* . The *tblastn* search of *D. melanogaster* JhI-26-PA (query) against the *D. dunni* (GenBank Accession: GCA_018152125.1 ) Genome Assembly database (subject) placed the putative ortholog of *JhI-26* within contig_344 ( JAECXC010000339 ) which corresponds to Augustus gene prediction JAECXC010000339 .g2190.t1 (E-value: 0.0; percent identity: 57.87%; query coverage: 97% as determined by *blastp* ). Immediately upstream of the putative ortholog is the Augustus gene prediction JAECXC010000339 .g2189.t1 which also corresponds to *JhI-26* (E-value: 1.00E-75; percent identity: 55.71%; query coverage: 96%, as determined by *blastp* . The putative ortholog and paralog are flanked upstream by the Augustus gene predictions JAECXC010000339 .g2188.t1 and JAECXC010000339 .g2187.t1, which correspond to *CG30324* and *CG7755* in *D. melanogaster* (E-value: 6.00E-66 and 1.00E-145; percent identity: 55.15% and 55.87%; query coverage: 100% and 99%, respectively, as determined by *blastp* ; Figure 1A ; Altschul et al., 1990). The putative ortholog of *JhI-26* is flanked downstream by the Augustus gene predictions JAECXC010000339 .g2191.t1 and JAECXC010000339 .g2192.t1, which correspond to *CG30100* and *CG7747* in *D. melanogaster* (E-value: 2.00E-68 and 0.0; percent identity: 73.68 and 84.72%; query coverage: 92% and 100% respectively, as determined by *blastp* ).

These results suggest that *JhI-26* underwent a duplication event in *D. dunni* or sometime earlier in the *immigrans-tripunctata* radiation of *Drosophila* . Scanlan et al. (2020) noted that the clade of EcKL that contains *JhI-26* is unstable and blooming ( *e.g.* , four gene duplication events identified). Based on our *blastp* results and the gene models built for each prediction, we hypothesize that Augustus gene prediction JAECXC010000339 .g2190.t1 represents the ortholog and JAECXC010000339 .g2189.t1 is a paralog. The putative ortholog assignment for *JhI-26* in *D. dunni* ( JAECXC010000339 .g2190.t1) is supported by the following evidence: The *tblastn* results are of good quality, and all isoforms found in *D. melanogaster* also appear to be present in *D. dunni* . The Spaln alignment (Iwata and Gotoh, 2012) of the *D. melanogaster* protein and the GeMoMa prediction based on the *D. melanogaster* transcript of *JhI-26* both map to this location. Gene expression data corresponds with each gene prediction of the *JhI-26* ortholog, paralog, and neighboring genes in *D. dunni* . The gene predictions surrounding the *JhI-26* ortholog and paralog are not fully conserved. *CG30100* and *CG7747* are both downstream of *JhI-26* in *D. melanogaster* but are the second and third genes. *CG30324* and *CG7755* are both upstream of *JhI-26* in *D. melanogaster* but are the second and fifth upstream genes respectively. We conclude that JAECXC010000339 .g2189.t1 is an ortholog of *JhI-26* in *D. dunni* ( Figure 1A ).

***Protein Model***

Both the *JhI-26* ortholog and paralog in *D. dunni* have 4 coding sequences (CDS) within the genome sequence. The first unique protein sequence (JhI-26-PA) is translated from 1 mRNA isoform (JhI-26-RA; Figure 1B ). Relative to the ortholog in *D. melanogaster* , the CDS number is not conserved but the protein isoform count is *.* The sequence of JhI-26-PA ortholog in *D. dunni* has 56.4% identity (72.6% similarity) with the protein-coding isoform JhI-26-PA in *D. melanogaster* , as determined by *blastp* ( Figure 1C ). This level of divergence is not surprising given that *D. dunni* and *D. melanogaster* belong to two separate subgenera ( *Drosophila* and *Sophophora* , respectively) that diverged approximately 45-60 MYA (Russo et al., 1995; Tamura et al., 2004; Obbard et al., 2012). The sequence of the JhI-26-PA paralog in *D. dunni* has 54.0% identity (71.7% similarity). The lower matches and absence of a Spaln alignment or GeMoMa prediction are why we conclude the prediction JAECXC010000339 .g2188.t1 is a paralog. Coordinates of these curated gene models are archived in the CaltechDATA repository (see “Extended Data” section below).

## Methods

The annotation methods used in this project are adapted from those described in Rele et al. (2023), which includes algorithms, database versions, and citations for the complete annotation process developed for the Pathways Project. The methods for the current project are detailed in brief below with notes on significant differences between this protocol and the one described in Rele et al. (2023). The students use the GEP instance of the UCSC Genome Browser v.435 ( https://gander.wustl.edu ; Kent et al., 2002; Raney et al., 2024) to examine the genomic neighborhood of their reference detoxification gene in the *D. melanogaster* genome assembly (Aug. 2014; BDGP Release 6 + ISO1 MT/dm6). Students obtain the protein sequence for the *D. melanogaster* target gene for a given isoform and use a *tblastn* search of the sequence against their target *Drosophila* species genome assembly ( *D. dunni* ( GCA_018152125.1 – Kim et al., 2021)) on the NCBI BLAST server ( https://blast.ncbi.nlm.nih.gov/Blast.cgi , Altschul et al., 1990) to identify the putative ortholog location. Students compare the genomic neighborhood of the putative ortholog to that of the reference gene in *D. melanogaster* . This local synteny analysis includes a minimum of two upstream and downstream genes relative to the potential ortholog. As no RefSeq protein data is available for these species, comparisons are based on gene predictions that correlate with gene expression data in the putative ortholog neighborhood. Using the multiple alignment tracks feature in the Genome Browser, students examine other sets of genomic evidence, including Spaln alignment of *D. melanogaster* proteins, multiple gene prediction tracks (e.g., GeMoMa, Augustus, NSCAN PASA-EST), and RNA-Seq mixed sex adult expression data from the target species generated by Erlenbach et al. (2023; https://doi.org/10.5061/dryad.hdr7sqvq2 ). Information on the genomic structure information (e.g., CDSs, intron-exon number, number of isoforms) for the reference gene in *D. melanogaster* is retrieved using Gene Record Finder ( https://gander.wustl.edu/~wilson/dmelgenerecord/index.html ; Rele et al *.,* 2023). To determine approximate splice sites within the target gene, a *tblastn* search using the CDSs from the *D. melanogaste* r reference gene against the putative ortholog location (10kb up- and downstream of the target gene prediction). Coordinates of the CDS(s) are refined by examining aligned RNA-Seq data, identifying canonical splice site sequences, and ensuring the maintenance of an open reading frame. Students confirm the biological validity of their target gene model using the FlySeq Gene Model Checker ( https://gander2.wustl.edu/~wilson/genechecker-flyseq/ ), which compares the hypothesized target gene model's structure and translated sequence against the *D. melanogaster* reference gene. At least two independent models for this gene are generated. These models are reconciled by a third independent researcher to produce the final model presented here. Note: comparison of 5' and 3' UTR sequence information is not included in this GEP CURE protocol.

## Data Availability

Description: Zipped archive containing FASTA, PEP, and GFF of ortholog and paralog. Resource Type: Model. DOI: https://doi.org/10.22002/bsxb1-1sx23
